# Molecular detection of a novel mutation in the *TPO* gene associated with congenital hypothyroidism in a cat: Case report

**DOI:** 10.5455/javar.2024.k854

**Published:** 2024-12-29

**Authors:** César Gallego-Munevar, Nicolas Carrillo-Godoy, Iang Schroniltgen Rondón-Barragán

**Affiliations:** Laboratory of Immunology and Molecular Biology, Faculty of Veterinary Medicine and Zootechnics, Universidad del Tolima, Ibagué-Tolima, Colombia

**Keywords:** Hypothyroidism, polymorphism, thyroperoxidase, sequencing, homozygous, heterozygous

## Abstract

**Objective:**

The objective of this study was to analyze the sequence of different fragments of the thyroperoxidase (TPO) gene from a cat diagnosed with congenital hypothyroidism (CH).

**Materials and Methods:**

The feline was diagnosed due to high serum concentrations of thyroid-stimulating hormone and low T4. The analysis of sequences containing mutations in the TPO gene from dogs with CH allowed for the prediction of mutation sites within the gene in an affected cat. In addition, the design of a polymerase chain reaction-based test allowed the amplification and sequencing of these gene segments. In addition, after the death of the patient, a necropsy and histopathology were performed, looking for macroscopic and microscopic alterations of affected organs.

**Results:**

The necropsy examination showed megacolon, cardiac concentric left ventricular hypertrophy, and bilateral enlargement of the thyroid gland. The histopathology of the thyroid showed follicular hypoplasia and low colloid production. gDNA analysis allowed the detection of mutation in the *TPO* gene, which corresponded to one transition in the nucleotide 12.542 (A > G) and heterozygous variations located in the nucleotide 14.627 (G/A) and in the nucleotide 30.713 (G/C).

**Conclusion:**

Due to the presence of these polymorphisms, it is suspected that one monoallelic expression of mutant alleles is present. More studies that allow an understanding of the role of the heterozygous in this pathology are required, as well as the role of gene mutations related to CH in cats. On the other hand, the data from the present study serve as the base for the development of a molecular test that allows a fast and accurate diagnosis of HC in cats.

## Introduction

Congenital hypothyroidism (CH) is a rare, underdiagnosed hormonal disorder in domestic animals, characterized by dysgenesis or dyshormonogenesis of the thyroid gland [1,2]. CH has been described in different species, including humans, dogs, and cats, and the clinical signs are closely related to a failure in the physiological functions of thyroid hormones, related to poor muscle and brain development, alterations in energy balance, developmental abnormalities such as disproportionate dwarfism, large head, flat face, smaller neck, body, and extremities, delayed tooth eruption, epiphyseal dysplasia, and delayed physeal closure, as well as the presence of palpable bilateral mobile goiters, bradycardia, hypothermia, swollen tongue, and flaccid muscle tone [2–4]. Furthermore, in humans, premature birth and low thyroid-stimulating hormone (TSH), and thyroxine (T4) exposure during gestation are often a cause of CH in the neonate [5]. However, this has not been described in companion animals.

In humans with CH, variants of some genes associated with the development of dysgenesis and dyshormonogenesis have been reported, the latter being inherited in a recessive manner [2], and related to mutations in genes such as TPO (thyroperoxidase), DUOX2, DUOXA2, SLC26A4, SLC5A5, TG and IYD [1,2,4]. In dogs and cats with CH, mutations have been described in some of these genes that have been shown to affect the synthesis and functions of the corresponding protein [1,2].

The diagnosis of CH is based on the clinical signs and measurement of the blood concentrations of T4 and TSH; in addition, scintigraphy can be used to differentiate between dysgenesis and dyshormonogenesis [2]. Due to its rare incidence and difficult diagnosis, many individuals die at birth or at an early stage of postnatal development before a specific treatment can be established [2]. Molecular tests are rarely used for the diagnosis of CH in domestic animals, and their use seems almost exclusive in research; however, they can help to understand the pathogenesis of CH [6]. Thus, the aim of this study was to report a clinical case of a cat with CH and to characterize the associated *TPO* gene mutations.

## Case Presentation

**Figure 1.**
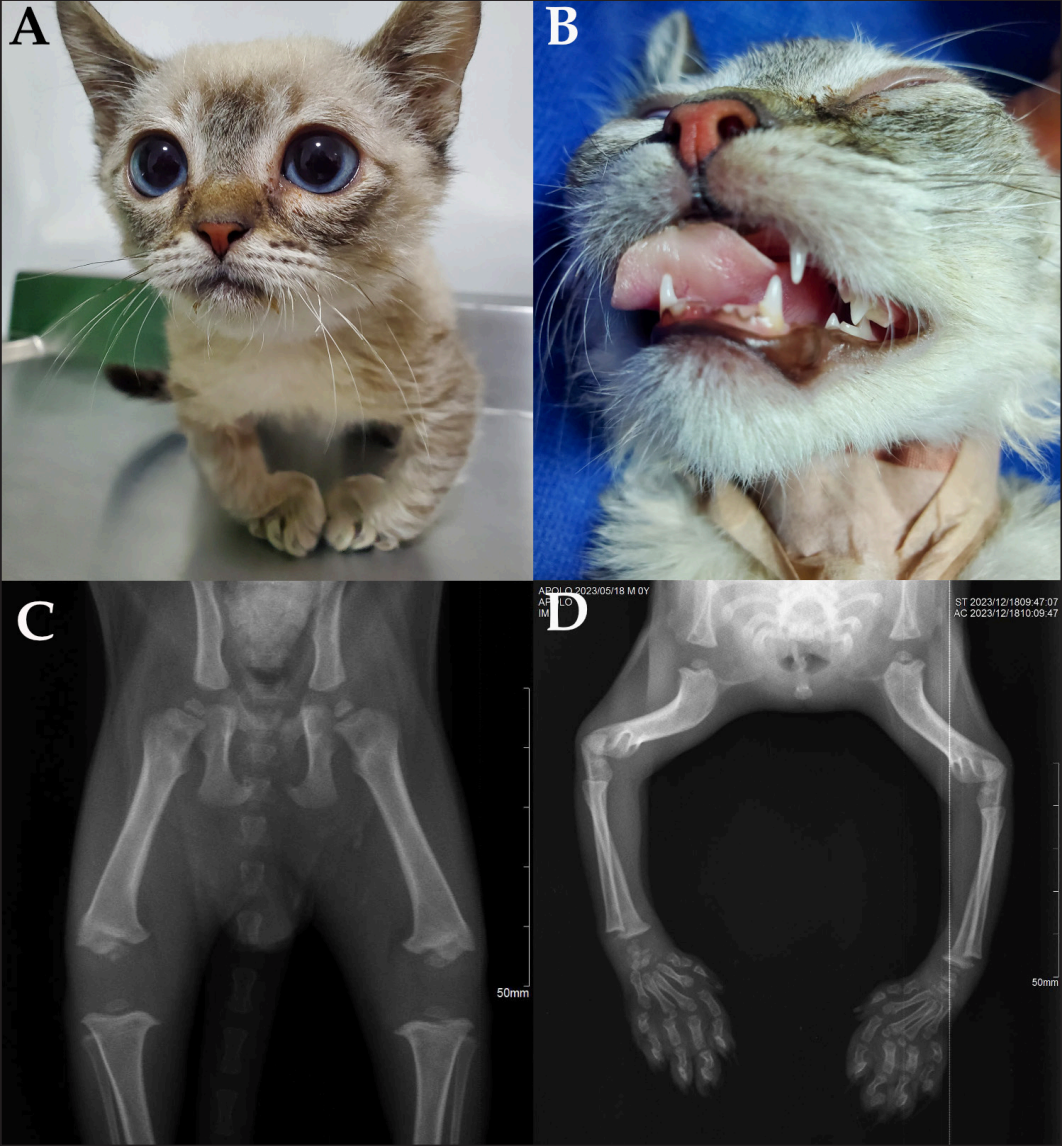
Feline patient, 7-month-old, showing developmental alterations, decrease in postnatal growth, wide head, larger eyes, osteodystrophy of forelimbs (A), delay in the shedding of teeth and absence in the eruption of decidual incisors (B). Radiographic study of the hip, ventrodorsal view showing hip dysgenesis with incomplete closure of the pelvis and the incomplete formation of the knee joint (C), forelimbs in cranial-caudal view showing epiphysial dysgenesis of the scapula-humerus joint, elbow joint and carpus-radio-ulnar joint (D).

A 7-month-old male cat was referred for consultation showing growth disorders such as dwarfism, delayed tooth shedding, and deforming osteodystrophy in thoracic limbs (Fig. 1). In addition, the patient showed hemoglobin concentration of 8.3 gm/l (reference interval 9.3–15.3 gm/l), the packed-cell volume of 26.3% (reference interval 28%–49%), blood urea nitrogen of 55.1 mg/dl (reference interval 4.5–23.5 mg/dl), and average temperature of 37°C. The measurement of IGF-1, T4, and TSH showed a concentration of 38 μg/dl (reference interval 200–800 μg/dl), 0.5 μg/dl (reference interval 0.8–4.7 μg/dl), and 0.82 UI/ml (reference interval 0.00–0.20 UI/ml), respectively, thus it was diagnosed with hypothyroidism, and the treatment was started with levothyroxine 20 μg/kg every 24 h.

Due to constipation problems, a radiographic study was performed showing megacolon as well as epiphyseal dysgenesis and delayed physeal closure (Fig. 1C and D). The patient was undergoing surgery two times to remove feces accumulated in the colon, which was associated with a failure of intestinal transit.

**Figure 2.**
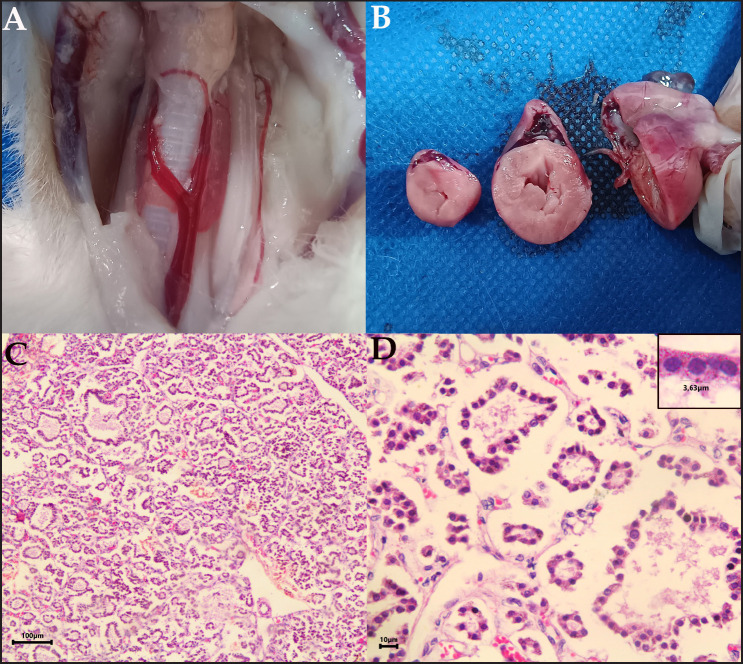
Necropsy examination of the feline patient with CH. Bilateral enlargement of thyroid gland (A). Cardiac concentric hypertrophy of left ventricle (B). Histopathological assessment of the thyroid gland. Thyroid follicles of several sizes and scarce concentration of colloid were detected (C). Follicles are hypoplastic, surrounded by loose connective tissue and few parafollicular cells, in addition, intracytoplasmic granules are evident in the thyrocytes (box) (D).

One week after performing the second enterotomy, the patient presented clinical complications that compromised their life. The necropsy examination showed the presence of megacolon, bilateral enlargement of the thyroid lobes (Fig. 2A), and cardiac concentric left ventricle hypertrophy (Fig. 2B). Samples were taken from the thyroid, heart, testicles, lungs, adrenal glands, hypophysis, thymus, and kidney for histopathology analysis.

The histopathology revealed follicular hypoplasia of the thyroid gland, with scarce colloid substance (Fig. 2C), a reduced number of parafollicular cells, loose connective tissue, and the presence of cytoplasmic granules in the follicular cells (Fig. 2D).

All the procedures, including the clinical examination and collection of samples, were approved by Act 11 of 2024 by the Bioethics Committee of The University of Tolima and were performed following the guidelines for animal welfare and research ethics based on the resolution number 8430/1993 and law 84/1989 and fulfilled the guidelines for animal care and use in clinical research and teaching.

### TPO gene analysis

Genomic DNA was extracted from a whole blood sample from the CH patient, as well as from a clinically healthy patient as a control, using the E.Z.N.A.^®^ Tissue DNA Kit (Omega Bio-tek, USA). DNA quality was verified by amplification of the beta actin (*actb*) gene and spectrophotometric measurement (Nano500, Allsheng, China).

An endpoint polymerase chain reaction (PCR) was carried out for the amplification of 3 regions of the *TPO* gene, which contained single nucleotide polymorphism (SNPs) previously reported in cats and dogs [2,7,8]. Primers were designed based on the sequence of the cat´s *TPO* gene (accession number NC_058370.1) (Table 1) in Geneious Prime v2024.0.5 software [9]. The PCR was performed in a Proflex^TM^ thermocycler (ThermoFisher Scientific, United States) using a final reaction volume of 25 μl, composed of 14,875 μl of deionized distilled water, 5 μl of 5x Colorless buffer (Promega, United States), 1 μl of dNTPs at 1.5 mM (Invitrogen, USA), 1 μl of each primer (forward and reverse), 1 μl MgCl_2_, 0.125 μl of GoTaq^®^ DNA Polymerase (Promega, United States) and 1 μl of gDNA, with the following profile thermal profile: initial pre-denaturation at 95°C for 3 min, followed by 35 cycles of denaturation at 95°C for 30 sec, annealing at 55°C for 30 sec and an extension at 72°C for 30 sec, followed by a final extension at 72°C for 5 min. The amplicons were revealed by horizontal electrophoresis in 2% agarose gel using the Hydragreen^®^ as DNA dye (ACTGene, United States) for 30 min at 100 volts (MyGel mini, Accuris, United States) and visualized under ultraviolet transilluminator (ENDURO GDS™, Labnet International, United States). Finally, the amplicons were purified and sequenced using the Sanger methodology (Macrogen Inc., South Korea). Sequence analysis was performed by Geneious Prime v2024.0.5 software [9], using the sequence NC_058370.1 as a reference. The sequences obtained were submitted to GenBank.

**Table 1.** Primer sequences for the amplification and sequencing of variable region of the *TPO* gene in domestic cat, *Felis catus*.

Gene	Reference	Sequence 5'-3'	Location	Tm (°C)	Amplicon (bp)
*TPO*	[7]	F- CAGCGAACGAGAGAGCCCTT	12.346–12.415	59.5	309
		R- AAAAGCCAAGTGTCGGTCCT	12.704–12.685	57	
	[2]	F - AGGGGATTGTTGAGAGAGCG	14.409–14.428	56.9	292
		R - CCCGTGATGAGCCTGTACTT	14.700–14.681	56.8	
	[8]	F - GTGTTTTCCTGCCACCACG	30.665–30.683	57.1	220
		R- TTTATTTCCCCGCCACCTGG	30.884–30.865	57.8	
*actb*	This study	F-GGCTACAGCTTCACCACCAC	-	60.96	497
		R-TACTCCTGCTTGCTGATCCACA	-	61.42	

**Figure 3.**
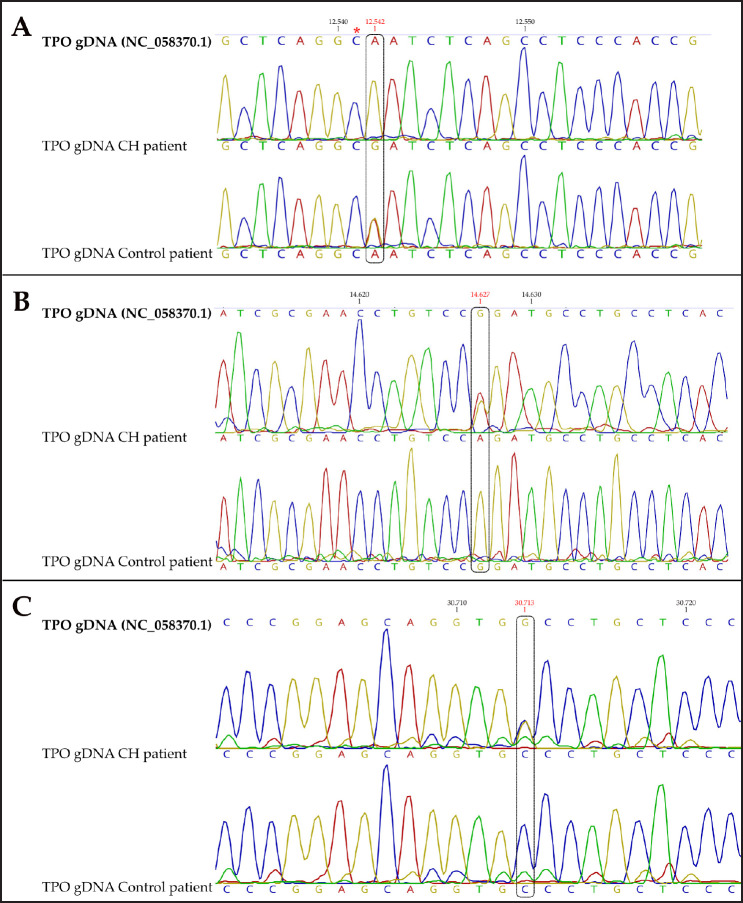
Variations in the sequence of the TPO gene from the feline patient with CH versus control patient. Reference *TPO* gene sequence was obtained from the GenBank, accession number NC_0583709.1. Variations in the sequence are indicated with a rectangle and the position number in red.

The comparison of the sequences showed a variation at nucleotide 12,542 (A > G), a position approximates that described in Toy Fox Terriers dogs [7] (Fig. 3A). The analysis of the sequence used as a control demonstrates a polymorphism (G/A) in this same position (Fig. 3A). It is also evident that the patient with CH is heterozygous for the variation previously described in cats by Van Poucke et al. [2] (Fig. 3B). Finally, the analysis of the fragment that contained the mutation site described in dogs by Major et al. [8] showed that the control patient presents a variation in nucleotide 30,713 (G > C), while the patient with CH presents a polymorphism at this position (Fig. 3C).

## Discussion

Hypothyroidism is a congenital or acquired endocrinopathy, which is usually common in dogs but rare in cats; however, it is classified as primary or central, depending on the hypothalamic-pituitary-thyroid axis condition [10]. Hypothyroidism of congenital origin has been described in humans and several animal species, with dyshormonogenesis being a common cause, related to failures in the synthesis and function of TPO, due to the inability in the organification of iodine [2–4,8,11,12].

The analysis of genomic sequences of canine *TPO* allowed us to identify the mutation sites described by Fyfe et al. [7] and Major et al. [8], and with the subsequent alignment of canine and feline sequences, detect conserved regions to predict possible mutations within the cat’s genome sequence. The sequencing of the different regions analyzed of *TPO* from the cat with CH demonstrated a transition from A > G, which creates a change in the codon encoding for Gln to one for Arg; in addition, the control patient presented polymorphism for this variation. Noteworthy, this variation was found in a homologous position to that reported by Fyfe et al. [7] in Toy Fox Terriers (Fig. 3A, asterisk), in which a C > T transition was demonstrated in dogs affected with CH, generating a premature stop codon that caused the translation of a nonfunctional truncated protein, and in addition, a polymorphism on the same variation was reported in healthy patients.

Furthermore, we demonstrated that the cat with CH showed a polymorphism (G/A) for the variation described by Van Poucke et al. [2], in contrast with the reported, since the affected animals were homozygous, which demonstrates that both homozygotes and heterozygotes for this variation may develop CH. This finding may be associated with the allele frequency of 9%, estimated previously [2].

The analysis of the genomic sequence of the *TPO* gene in a French bulldog allowed us to identify a homozygous T > C transition in position +2 of the splice donor site of intron 12. This variation generated a protein coming from an ORF 171 bp shorter, corresponding to the exact loss of exon 12 [8]. The region of the variation described by Major et al. [8] shares a high identity with the region of the cat *TPO* gene of our study, which allowed the analysis of this sequence, despite the absence of homologous mutations with French bulldogs. A G/C polymorphism was identified towards the 5’ end of the analyzed region, which changes the codon encoding for Pro to a codon for Ala.

The analysis of *TPO* variants in animals and humans with CH demonstrates that CH is an autosomal recessive disorder, since parents are obligate carriers of polymorphisms in the *TPO* genetic sequence and the affected offspring are homozygous for the mutant allele [2,4,7,8,12]. Therefore, animals and humans heterozygous for a variation in *TPO* do not usually develop clinical signs compatible with CH; however, in this study, we identified two heterozygous variations in *TPO* of the affected cat, and the presence of clinical signs in this patient may be associated with a monoallelic expression of the mutant *TPO* allele due to the dominant-negative effect, of which there are few reports in humans [13,14]. Furthermore, it is possible that CH in this case is inherited in an autosomal dominant manner [15]. Another homozygous mutation was also identified that possibly contributes to the poor production or function of *TPO* in the affected cat. For all of the above, this unusual genotypic presentation could lead to a variable phenotypic manifestation in carrier animals, similar to what was described in humans with a single heterozygous variation of *TPO* that led to a mild to moderate phenotype in patients with CH [15]. However, there is no obvious correlation between genotype and phenotype for *TPO* mutations [4], and further studies in cats are needed to understand the pathogenesis of heterozygous *TPO* variations.

The analysis of a single gene is a limitation in this study, because more than one gene can participate in the CH process in an individual, and the study of individual genes provides restricted information to find a relationship between genotype and phenotype [8]. Therefore, it is necessary to explore the comprehensive effect of all CH-related genes to reveal the genetic complexity that the interaction between multiple affected genes may have. Furthermore, somatic gDNA and cDNA analysis of thyroid tissue was not performed in our study, which allowed to demonstrate monoallelic expression in human patients with heterozygous mutations [14].

The genetic alterations observed may be related to the clinical presentation and postmortem findings. The necropsy revealed bilateral enlargement of the thyroid, a pathology that has already been described before in patients with CH [2,7,8,12] and has been related to constant stimulation of high concentrations of TSH dependent on the amount of hormone produced and the amount of iodine transferred in the thyroid gland [7]. Furthermore, CH can develop goiter due to dyshormonogenesis due to genetic alterations that alter the ability to concentrate iodine, defects in the organization and synthesis of TG, and supplementation with levothyroxine in conventional doses may not be sufficient to reduce the size of the gland [12], which may explain the poor response to treatment in the affected cat in the present study. According to studies carried out in Toy Fox Terriers and Belgian cats [2,7], goiter in these animals was related to mutations in *TPO*, so the homology between these studies and the present case allows us to deduce that the enlargement of the thyroid may be due to defects in the organization of iodine.

Despite the above, goiter was not evident, and the histopathological alterations, in this case, differed from those reported in dogs with CH that developed goiter, in which follicular epithelial hyperplasia, transition to a columnar morphology of the follicular epithelium, and few colloids were evident, and follicles of different sizes separated by widened collagen septa [7,8,12]. In rats with CH, goiter, and hyperplasia are necessary to compensate for the deficient production of T4, which is synthesized by the release of TG into the follicular lumen from dead hypertrophic thyrocytes whose number remains constant due to the hyperplasia [16]. Therefore, follicular hypoplasia in the affected cat may be related to a poor response to TSH stimulation that prevents the proliferation of thyrocytes that generate an evident goiter and therefore the synthesis of adequate concentrations of T4.

On the other hand, low T4 concentrations in affected cats may be related to left ventricular hypertrophy, as reported in dogs with CH, in which dilated cardiomyopathy was observed [1]. This may be explained by an insufficient expression of proteins involved in muscle contraction, since in rats the expression of phospholamban is regulated by thyroid hormones [17]; furthermore, in humans with hypothyroidism, thyroid hormone receptors at the level of the myocardium are underregulated [18]. However, these mechanisms have not been clarified in cats.

The presence of megacolon in the affected cat is a poorly documented condition in animals, which has been described more frequently in humans with CH [4]; therefore, constipation problems in newborn cats may also help lead to the clinical condition toward the diagnosis of CH.

Although clinical manifestations and paraclinical tests allowed the diagnosis of hypothyroidism, molecular tests helped clarify the congenital condition of endocrinopathy; therefore, molecular techniques represent a tool to understand CH and a diagnostic alternative for affected patients. Molecular assays for the detection of *TPO* mutations have been standardized in some dog breeds [7]. Furthermore, in some countries the use of diagnostic tests for CH in human newborns is common [4], allowing early detection and treatment. High-resolution melting curve analysis has made it possible to identify mutations in the *TPO* gene, as well as to discriminate between heterozygous and homozygous carriers [6], which overcomes problems of biochemical tests, such as the detection of maternal TSH in newborns. Therefore, by providing a faster, more affordable, and reliable mutation detection method, PCR- and *ngs*-based approaches will be able to improve diagnosis and treatment selection, as well as being simple, accurate, and cost-effective.

## Conclusion

In the present report, we showed for the first time the presence of three variations of the *TPO* gene in a cat with CH, including polymorphisms that may be related to the development of the disease. Nevertheless, the understanding of CH requires the study of several genes that may play a role in the origin and clinical form. Molecular characterization can be an important tool for an accurate and fast diagnosis.

## List of abbreviations

A, adenine; *actb*, beta actin; C, cytosine; CH, congenital hypothyroidism; DNA, deoxyribonucleic acid; dNTP, deoxyribonucleotide triphosphate; G, guanine; PCR, polymerase chain reaction; SLC26A4, solute carrier family 26 member 4; *SLC5A5, *solute carrier family 5 member 5; T, thymine; T4, tetraiodothyronine; TG, thyroglobulin; TPO, thyroperoxidase; TSH, thyroid stimulating hormone.
